# Research progress of MRI-based radiomics in hepatocellular carcinoma

**DOI:** 10.3389/fonc.2025.1420599

**Published:** 2025-02-06

**Authors:** Xiao-Yun Xie, Rong Chen

**Affiliations:** ^1^ Department of Radiation Oncology, Medical School of Southeast University, Nanjing, China; ^2^ Department of Radiation Oncology, Zhongda Hospital, Nanjing, China

**Keywords:** MRI, radiomics, hepatocellular carcinoma, machine learning, treatment, diagnosis

## Abstract

**Background:**

Primary liver cancer (PLC), notably hepatocellular carcinoma (HCC), stands as a formidable global health challenge, ranking as the sixth most prevalent malignant tumor and the third leading cause of cancer-related deaths. HCC presents a daunting clinical landscape characterized by nonspecific early symptoms and late-stage detection, contributing to its poor prognosis. Moreover, the limited efficacy of existing treatments and high recurrence rates post-surgery compound the challenges in managing this disease. While histopathologic examination remains the cornerstone for HCC diagnosis, its utility in guiding preoperative decisions is constrained. Radiomics, an emerging field, harnesses high-throughput imaging data, encompassing shape, texture, and intensity features, alongside clinical parameters, to elucidate disease characteristics through advanced computational techniques such as machine learning and statistical modeling. MRI radiomics specifically holds significant importance in the diagnosis and treatment of hepatocellular carcinoma (HCC).

**Objective:**

This study aims to evaluate the methodology of radiomics and delineate the clinical advancements facilitated by MRI-based radiomics in the realm of hepatocellular carcinoma diagnosis and treatment.

**Methods:**

A systematic review of the literature was conducted, encompassing peer-reviewed articles published between July 2018 and Jan 2025, sourced from PubMed and Google Scholar. Key search terms included Hepatocellular carcinoma, HCC, Liver cancer, Magnetic resonance imaging, MRI, radiomics, deep learning, machine learning, and artificial intelligence.

**Results:**

A comprehensive analysis of 93 articles underscores the efficacy of MRI radiomics, a noninvasive imaging analysis modality, across various facets of HCC management. These encompass tumor differentiation, subtype classification, histopathological grading, prediction of microvascular invasion (MVI), assessment of treatment response, early recurrence prognostication, and metastasis prediction.

**Conclusion:**

MRI radiomics emerges as a promising adjunctive tool for early HCC detection and personalized preoperative decision-making, with the overarching goal of optimizing patient outcomes. Nevertheless, the current lack of interpretability within the field underscores the imperative for continued research and validation efforts.

## 1 Introduction

Primary liver cancer (PLC) remains a significant global health burden, ranking as the sixth most common malignant tumor and the third leading cause of cancer-related mortality worldwide (1). In China, where liver cancer prevalence is particularly pronounced, incidence and mortality rates reached 18.3/100,000 and 17.1/100,000, respectively, in 2018. Projections suggest a concerning upward trend, with anticipated increases of 50.5% and 54.9% in new cases and deaths by 2040, respectively (2, 3). Hepatocellular carcinoma (HCC) stands as the predominant form of PLC, representing 70% to 85% of cases. Chronic liver disease and liver cirrhosis are primary risk factors contributing to its development (4). Alpha-fetoprotein (AFP) and liver ultrasound (US) are the most widely used methods for HCC screening (5). The diagnosis and staging of HCC involve various imaging examinations, including US, CT, and MRI. However, due to HCC’s insidious onset and nonspecific early symptoms, diagnosis often occurs at an advanced stage, limiting the efficacy of potentially curative interventions and contributing to the high recurrence rates post-surgery (6, 7). There exists a pressing need for non-invasive methodologies capable of predicting tumor histopathological characteristics, treatment response, and recurrence rates to enhance early diagnosis rates and monitor treatment efficacy effectively.

Thus, we introduce the concept of radiomics. Radiomic, proposed by Dutch scholar Lambin in 2012 (8), harnesses high-throughput extraction of imaging features, including shape, texture, and intensity, to elucidate underlying tissue physiology and pathology. By integrating these features with clinical data and employing artificial intelligence (AI) models, radiomics adeptly addresses the challenges posed by tumor heterogeneity, offering promising avenues for tumor diagnosis, treatment, and prognosis analysis, thereby improving patient survival rates and facilitating personalized medicine. MRI, with its distinctive advantages in radiomics such as high soft tissue contrast, absence of ionizing radiation, and multi-parametric imaging capabilities, emerges as a pivotal modality for HCC assessment. It enables comprehensive visualization of tumor morphology, boundaries, internal structure, and vascular relationships critical for surgical planning and prognosis assessment (9). Meta-analyses consistently underscore MRI’s superior sensitivity for HCC diagnosis compared to CT (10–12). This review consolidates current research progress on MRI-based radiomics in hepatocellular carcinoma, examining its potential applications and future directions in clinical practice.

## 2 Methods

**Figure 1:**
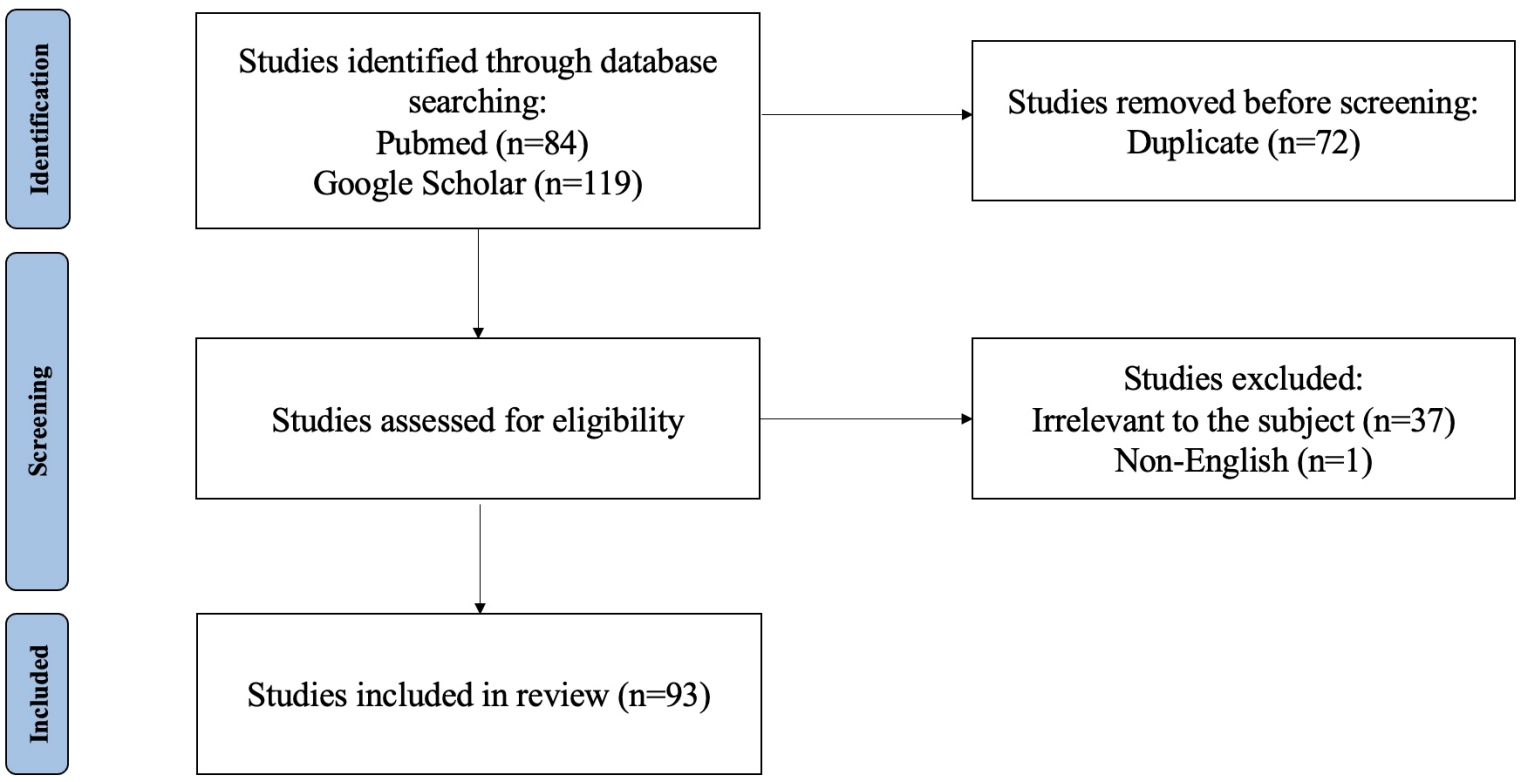
Flow diagram of study selection.

This study presents a comprehensive literature review aimed at elucidating recent advancements in MRI-based radiomics within the context of hepatocellular carcinoma (HCC). The review was conducted through searches of PubMed and Google Scholar databases. Key search terms included hepatocellular carcinoma, HCC, liver cancer, magnetic resonance imaging, MRI, radiomics, deep learning, machine learning, and artificial intelligence. The inclusion criteria were as follows: ① original research and review published between July 2018 and Jan 2025; ② literature related to MRI radiomics or AI; ③literature related to diagnosis and differentiation, histological grading, microvascular invasion (MVI) assessment, surgery, ablation, transarterial chemoembolization (TACE), external beam radiotherapy (EBRT), systematic therapy efficacy, adverse effects of therapy, recurrence, and metastasis. Exclusion criteria comprised non-English publications, articles unrelated to the designated topic, those predating June 2018 or extending beyond Jan 2025, as well as duplicate entries across both databases. In total, 182 articles were retrieved. After screening the titles, abstracts, and full texts, only 88 papers met the inclusion criteria (**Figure 1**).

The PDFs of these 88 articles were imported into Zotero, and two authors (Xie Xiaoyun, Chen Rong) independently reviewed the eligibility of all papers and manually tagged them in Zotero. The studies were categorized into eight groups: diagnostic typing, pathological grading, microvascular invasion (MVI), surgical prognosis, TACE prognosis, targeted therapy and immunotherapy prognosis, adverse reactions, and recurrence and distant metastasis. Two authors independently extracted important information such as authors, year, number of study subjects, outcome indicators, methods for establishing models, and model efficacy using standardized forms, and discrepancies were resolved by consensus (**Table 1**).

**Table 1:** Representative studies evaluating MRI radiomics tools for treatment slection of hepatocellular carcinoma.

Author	Year	Modality	Treatment	Subject number (Train/ Valid)	Specific Outcome	Model	Best performance
Zhang Z (45)	2022	T2WI, DWI	surgery	83/37	OS	LASSO	C-index 0.92
Wang Y (46)	2023	AP, PVP, T2MI	locoregional treatment	70/30	Response/Non-response	LASSO	AUC 0.867
Aujay G (48)	2022	AP, PVP	TARE	22	Response/Non-response	LR	AUC 1
Zhao Y (49)	2023	AP, PVP, DP	TACE	96/42	Response/Non-response	LR	AUC O.918
Zhao Y (50)	2021	AP, PVP, DP	TACE	85/37	Response/Non-response	LR	AUC 0.878
Zhao Y (51)	2023	T2WI	HAIC	79/33	PFS	LASSO	AUC 0.79
Sun Y (52)	2020	T2WI, DWI	TACE	67/17	PD/NPD	LASSO	AUC 0.786
Ym H (54)	2022	CT	RT	105/26	OS	LASSO	C-index 0.79
Gong XQ (56)	2023	T1WI, T2WI	PD-1/PD-L1	70/38	Response/Non-response	LASSO	AUC 0.898
Chen Y (57)	2021	MRI	PHLF	111/33	PHLF	LR	AUC 0.956
Wang Q (58)	2023	TIWI	PHLF	276	PHLF	Machine learning	OR(subgroup1: subroup2) 2.83:2.41
Shen PC (59)	2022	CT	RILD	50/34	RILD	RF, LR	RF: F1 score 0.857, sensitivity100, specificity 93.3%, accuracy 94.4% LR: F1 score 0.8, sensitivity 66.7, specificity 100, accuracy 94.4%

## 3 Results

### 3.1 Work process of radiomics

Radiomics, an innovative approach in medical imaging analysis, entails a systematic workflow for extracting comprehensive quantitative information from imaging data. The fundamental steps involved in radiomics are as follows (13–15):

#### 3.1.1 Image acquisition

Radiomics analyses demand high-quality image data acquired under standardized conditions. Adherence to standardized MRI scanning parameters, encompassing slice thickness and sequences, is imperative to facilitate consistent radiomics analysis. Additionally, preprocessing steps are essential to ensure data accuracy and consistency, involving contrast adjustment, standardization of image parameters, denoising, and correction of scanning artifacts.

#### 3.1.2 Image segmentation

A precise and repeatable image segmentation process is necessary for radiomics analysis, using manual, semi-automated, or fully automatic image segmentation software to sketch tumors or normal tissues as the region of interest (ROI) after standardized medical imaging data has been obtained. ROI refers to a specific area of particular interest and analysis in image analysis. Software like MIM, ITK-SNAP, 3DSlicer, and ImageJ are frequently utilized. Manual segmentation has the advantage of high precision but may be disrupted by subjective factors, which is suitable for lesions with difficult-to-detect boundaries. Semi-automatic or automatic segmentation methods are highly reproducible and meet the requirements for massive data segmentation for tumors with regular shapes and clear boundaries (16).

#### 3.1.3 Feature extraction

The core step of radiomics is the extraction of high-throughput radiomics features. Commonly used radiomics features include ① morphological features, such as the location, shape, size, blood vessel distribution, and whether there are burrs and necrosis of the tumor. ② First-order grey scale histogram features are statistical characteristics that describe the distribution of image gray levels, including maximum, minimum, mean, standard deviation, variance, and so on. ③ Texture features at the second and higher orders illustrate the link between the spatial distribution of grey values in an image. The greyscale covariance matrix and grey scale tour length matrix are examples of second-order texture feature methods. Grey level region size matrix and neighborhood grey level difference matrix are examples of higher-order texture feature methods. ④ Filter and transform-based features. Commonly used software for feature extraction includes IBEX, MaZda, Pyradiomics, and CERR. Using different software in conjunction with each other can help obtain more comprehensive radiomics features from images (17).

#### 3.1.4 Feature selection

Given the potentially vast number of extracted features, feature selection and dimensionality reduction are imperative to enhance model predictivity. The LASSO Cox regression model is the most commonly used method. After feature selection, the final number of radiomics features generally ranges from a few to several tens.

#### 3.1.5 Model establishment

Model building and prediction are breakthrough points in radiomics analysis, serving as auxiliary tools for diagnosis and efficacy prediction. In radiomics analysis, univariate analysis often does not yield reliable results, thus machine learning algorithms are typically used to establish classification or prediction models, such as support vector machine (SVM), random forest, gradient boosting machine (GBM), or deep learning networks. The goal of the model is to assist in early diagnosis or predict clinical outcomes of hepatocellular carcinoma based on imaging features, such as survival rate, recurrence risk, or treatment response.

#### 3.1.6 Model validation

The performance of radiomics models necessitates rigorous evaluation through internal and external validation to ensure their generalizability and clinical utility. Internal validation techniques include cross-validation. Cross-validation is a technique for assessing model performance by dividing the data into multiple subsets and using one subset as the test set while the rest serve as the training set in turn. While external validation involves testing the model on independent patient cohorts. Only validated models demonstrating high effectiveness are deemed suitable for clinical application.

### 3.2 MRI-based deep learning for HCC

In recent years, with the advancement of AI technology, we often rely on machine learning algorithms when constructing radiomics models. Deep learning is a subfield of machine learning that involves the use of artificial neural networks to process large amounts of data through multiple layers of neurons. In medical imaging, CNN is commonly used in radiological AI approaches for analyzing image data. The development of CNN in the medical field appeared almost at the same time as radiomics, and their progress can bring complementarity to both sides, improving the clinical applicability and universality of AI (18). There are also other deep learning methods that we will not discuss in this review for the time being. Currently, the application of deep learning is a hot topic in the field of radiomics. Current research indicates that there is a high level of heterogeneity among studies in the field of radiomics using deep learning, with significant variations noted in methodology, terminology, and outcome measures. This could lead to an overestimation of the diagnostic accuracy of DL algorithms in medical imaging. Despite this, deep learning still holds tremendous potential in the field of radiomics due to its significant advantages in image analysis. There is an immediate need for the development of artificial intelligence-specific EQUATOR guidelines, particularly STARD, in order to provide guidance around key issues in this field (19).

### 3.3 Clinical applications of MRI-based radiomics in hepatocellular carcinoma

MRI radiomics has a wide range of clinical applications, including assisting in differential diagnosis, subtype classification, pathological tissue grading, guiding the selection of treatment plans, and predicting the possibility of recurrence and distant metastasis. The specific details will be described in the following text.

#### 3.3.1 Differential diagnosis and subtype classification

Liver puncture biopsy is still the gold standard for HCC preoperative diagnosis, however, due to the high heterogeneity of tumors, biopsy cannot comprehensively assess the tumor, meanwhile, repeated operations will carry a higher risk of complications. Currently, imaging techniques including MRI, CT, and US are widely used to diagnose HCC. As we know, radiologists have a strong subjective effect on how images are interpreted. Therefore, a more objective method, such as MRI radiomics, is needed to improve diagnostic accuracy. ZHAO et al. (20) successfully established and validated a radiomics model based on contrast-enhanced MRI (CE-MRI) images for preoperative differentiation of fat-poor angiomyolipoma (fp-AML) and hepatocellular carcinoma (HCC) in non-cirrhotic patients. GAO et al. (21) developed a radiomics model based on unenhanced MRI images to distinguish small hepatocellular carcinoma (S-HCC) (≤2 cm) and pre-hepatocellular carcinoma (Pre-HCC), whose diagnostic performance significantly higher than that of radiologists (AUC = 0.518, p<0.05). HUANG et al.’s study (22) demonstrated that radiomics features extracted from Gd-EOB-DTPA-enhanced MR images could be used to diagnose preoperative dual-phenotype hepatocellular carcinoma (dual-phenotype hepatocellular carcinoma, DPHCC) with positive CK7 and CK19.

MRI radiomics not only can differentiate early-stage tumors from other diseases and distinguish between benign and malignant liver lesions, it also helps distinguish subtypes of primary liver cancer. The team led by ZHANG (23) established radiomics models based on T2WI, AP, PVP, T2WI + AP, T2WI + PVP, AP + PVP, and T2WI + AP + PVP sequences, with AUC values of 0.768, 0.838, 0.778, 0.880, 0.818, 0.832, and 0.884, respectively, demonstrating radiomics’ excellent ability to distinguish HCC from non-HCC. Similar models have also been developed by groups headed by LIU et al. (24), HUANG et al. (25), and ZHANG et al. (23) to assist physicians in non-invasively differentiating intrahepatic cholangiocarcinoma (ICC) from hepatocellular carcinoma (HCC) before surgery. Macrotrabecular-massive-type hepatocellular carcinoma (MTM-HCC) is a highly aggressive type of hepatocellular carcinoma, often indicating a poor prognosis. Zhu et al.’s (26) study successfully constructed a predictive model based on enhanced MRI radiomics to preoperatively predict MTM-HCC, aiding in early identification and improving patient prognosis.

In recent years, DL technology has been developed and has achieved excellent performance in the classification of hepatic lesions. Hamm CA et al. (27) developed a proof-of concept convolutional neural network (CNN)-based DL system and classified 494 hepatic lesions from six categories on MRI. The system demonstrated 92% accuracy, 92% sensitivity and 98% specificity, and their results showed a 90% sensitivity for classifying HCC compared to 60%/70% for radiologists.

#### 3.3.2 Histopathological grading

Accurate histopathological grading is pivotal for guiding treatment decisions in HCC patients. Its criteria are mainly based on tumor size, number of tumors, degree of differentiation, tumor growth pattern (such as whether it invades the capsule and the vascular), presence of microvascular invasion (MVI), and presence of satellite nodules. MRI-based radiomics features can reflect tumor biological characteristics, such as cell differentiation, invasiveness, and vascular invasion, which are crucial for tumor grading and staging, helping guide treatment plan selection. Hu et al. (28) constructed a radiomics model based on Gd-EOB-DTPA-enhanced MRI for distinguishing different histopathological grades of HCC, with an AUC of 0.71 (95% CI: 0.59–0.82) in the external validation set, indicating the potential of radiomics in preoperative prediction of HCC differentiation levels. Yan et al. (29) used Gd-EOB-DTPA-enhanced MRI data to extract radiomics characteristics. To find the best features, they successively used three feature selection techniques: Recursive Feature Elimination with Cross-Validation (RFECV), SelectFromModel (SFM), and SelectPercentile (SP). The combined model they developed, which integrates radiomics features and clinical predictive factors, demonstrated excellent performance in evaluating the grade of HCC in the test dataset (AUC: 0.801). Additionally, multiple studies by HAN et al. (30) and other researchers (31, 32) have also demonstrated that the radiomics models based on Gd-EOB-DTPA-enhanced or unenhanced MRI work well in preoperative assessment of HCC grading.

Among them, microvascular invasion (MVI) is an important pathological feature of HCC. which is defined as tumor cells infiltrating endothelium-lined vascular spaces, including microscopic vessels of the portal vein, hepatic artery, and lymphatic vessels. It is difficult to detect in routine imaging, as a result, its diagnosis primarily depends on postoperative histological examination (33). According to some studies (34, 35), MVI affects 15%–57% of patients with HCC and may be regarded as a tumor evolution process involved in the intrahepatic recurrence phase. It is one of the strongest predictors of HCC recurrence after liver transplantation or hepatectomy and a key risk factor for overall survival (OS) and recurrence-free survival (RFS) of HCC patients after treatment. Therefore, accurate preoperative prediction of MVI in liver cancer is of great significance, helping clinicians adjust treatment strategies promptly and improve patient prognosis.

**Figure 2:**
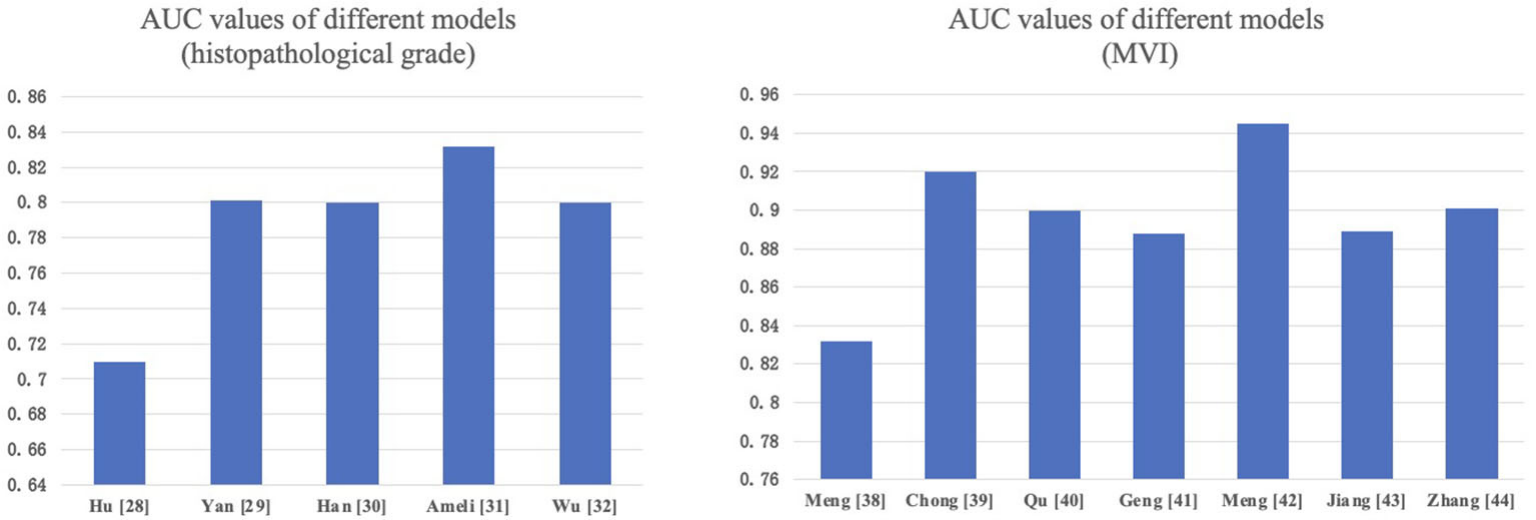
Comparison of the performance of different models.

Numerous academics have developed models, and meta-analyses (36, 37) have demonstrated that MRI radiomics has the potential for preoperative prediction of MVI in HCC. Meng et al. (38) showed that CT and MRI have comparable predictive performance for MVI in solitary HCC, but for HCC between 2-5 cm, MRI has a significant advantage over CT in predicting MVI. Chong et al. (39) and Qu et al. (40) retrospectively analyzed HCC ≤ 5cm patients and extracted Gd-EOB-DTPA-enhanced MRI radiomics features to construct predictive models, showing good performance. Geng et al.’s MRI radiomics model (41) demonstrated exceptionally high effectiveness (AUC=0.948). Meng et al. (42) extracted radiomics features from multimodal MRI images, including arterial phase (AP), delayed phase (DP), diffusion-weighted imaging (DWI), and fat-suppressed T2-weighted imaging (T2WI-FS) images. The cross-modality tensor fusion (CMTF) model they developed showed better AUC values compared to single-modality models. Jiang et al. (43) and Zhang et al. (44) also established predictive models for MVI in HCC patients before surgery based on multi-parametric MRI, proving the feasibility of multi-parametric MRI radiomics in predicting MVI. We compared the performance of various models in predicting HCC pathological grading and MVI in **Figure 2**.

#### 3.3.3 Treatment selection

At present, the stage of the tumor at diagnosis has a significant impact on the therapy option for HCC. As the Barcelona Clinic Liver Cancer (BCLC) system recommended, resection, transplantation, or local ablation are suitable for HCC patients at BCLC stages 0 or A. For BCLC stage B patients with good liver function, transarterial chemoembolization (TACE) is the first-choice treatment method. However, some patients may not benefit from TACE, so systemic therapy can be adopted as an alternative. While systemic therapy is the first-choice therapeutic option for people with BCLC stage C. However, treatment options for HCC vary in clinical practice, and because of tumor heterogeneity, patients can have drastically variable outcomes even within the same BCLC stage. Under these circumstances, MRI radiomics can predict treatment efficacy and patient prognosis by analyzing tumor morphological, textural, and intensity features, helping to assess treatment indications and formulate personalized treatment plans.

##### 3.3.3.1 Surgical resection and liver transplantation

MRI radiomics features can help predict the efficacy of surgery. Zhang et al. (45) combined clinical predictors to construct an MRI radiomics model that effectively works in predicting the overall survival (OS) of HCC patients after surgery. They included 120 patients and successfully constructed a model using features from T2WI and DWI images with LASSO regression.

##### 3.3.3.2 Local treatment

Local treatment is the preferred treatment for selected unresectable or inoperable liver-limited diseases, including ablation, arterial interventional treatment, or external beam radiation therapy (EBRT). Wang et al. (46) constructed a model based on MRI radiomics features and clinical factors to predict the efficacy of local treatment in hepatocellular carcinoma patients, helping further treatment planning.

Local ablation treatment involves techniques such as radiofrequency ablation (RFA), microwave ablation (MWA), and cryoablation, which can induce tumor necrosis. MRI radiomics can potentially aid in assessing the adequacy of the ablation area and predicting the risk of tumor residue or recurrence after ablation, but further improvement is still needed in current radiomics research on local ablation treatment.

Arterial interventional treatment includes TAE, TACE, DEB-TACE, and SIRT/TARE using Y-90 microspheres. For advanced liver cancer patients, TACE is often used as the first-line treatment. However, the efficacy of TACE in treating hepatocellular carcinoma is not stable, with a complete necrosis rate of only 22% to 29% (47), therefore accurately predicting the efficacy before TACE is crucial for treatment selection. Aujay et al. (48) used MRI radiomics data to evaluate the response of locally advanced hepatocellular carcinoma (HCC) patients to 90Y transarterial radioembolization (TARE) treatment, and the predictive model constructed with four radiomics features achieved ideal results. Zhao et al. (49, 50) used contrast-enhanced MRI (CE-MRI) tumor and peritumoral radiomics features to establish multiple models for preoperative prediction of HCC patient response to TACE treatment, aiding in personalized follow-up and further treatment strategy guidance. They also (51) combined clinical factors such as the albumin-bilirubin (ALBI) score to construct a radiomics predictive model, which also showed good efficacy and could serve as a biomarker to predict the treatment effect of unresectable HCC after HAIC. While Sun et al. (52) believed that clinical factors made no significant difference in the model’s efficacy. Additionally, Kang et al. (53) created a model that predicts how unresectable hepatocellular carcinoma (HCC) will react to transarterial chemoembolization in conjunction with molecular targeted therapy and immunotherapy by combining preoperative multi-parametric magnetic resonance imaging (MRI) radiomics features with clinical features (alpha-fetoprotein and neutrophil-to-lymphocyte ratio). With AUC and 95% CIs for treatment response prediction in the training cohort and two external validation cohorts of 0.956 (0.920-0.984), 0.895 (0.810-0.967), and 0.892 (0.804-0.957), respectively, the clinical-radiomics combined model demonstrated the highest efficacy among them. Radiotherapy options for unresectable HCC include external beam radiotherapy (EBRT) and stereotactic body radiotherapy (SBRT). In recent years, SBRT technology has rapidly developed, making radiotherapy an increasingly important treatment option for unresectable HCC. MRI radiomics can predict radiotherapy efficacy through tumor radiomics features and radiotherapy plans, guiding the selection of clinical treatment plans. Huang et al. (54) studied HCC patients with portal vein tumor thrombosis (PVTT) receiving radiotherapy, extracted two radiomics features, established an overall survival (OS) prediction model based on radiomics features, clinical characteristics, and radiotherapy (RT) dose parameters, and finally proving its predictive accuracy. Current studies are mostly based on CT radiomics, and the effect of MRI radiomics in predicting HCC radiotherapy efficacy remains to be further explored.

##### 3.3.3.3 Systemic therapy

The utilization of MRI radiomics features holds potential in the prediction of hepatocellular carcinoma (HCC) patient responses to chemotherapy, targeted therapy, and immunotherapy, hence facilitating the identification of optimal medications and treatment strategies. Yu et al. (55) assessed the efficacy of sorafenib and NK cell immunotherapy in HCC patients using MRI radiomics. They found a significant association between MRI characteristics and tissue biomarkers. Gong et al. (56) demonstrated that a radiomics model utilizing multi-sequence MRI has the capability to forecast the expression of PD-1 and PD-L1 before surgery in patients with hepatocellular carcinoma (HCC). This model has the potential to serve as an imaging biomarker for immune checkpoint inhibitor (ICI) therapy.

##### 3.3.3.4 Adverse reactions induced by treatments

Adverse responses are observed in all therapies. By accurately forecasting the likelihood and intensity of severe adverse reactions before treatment, we can enhance our capacity to make informed treatment choices and minimize the occurrence of serious adverse reactions. Chen et al. (57) investigated the occurrence of liver failure after surgery in patients with hepatocellular carcinoma (HCC). They developed a predictive model for posthepatectomy liver failure (PHLF) using Gd-EOB-DTPA-enhanced MRI radiomics. Wang et al. (58) employed unsupervised learning algorithms to develop a model capable of detecting high-risk patients for postoperative liver failure (PHLF) by utilizing preoperative Gd-EOB-DTPA-enhanced MRI radiomics characteristics. Shen et al. (59) predicted the risk of radiation-induced liver disease (RILD) in HCC patients after stereotactic body radiotherapy (SBRT) based on CT radiomics, including five predictors such as albumin-bilirubin grade, difference means, intensity, V5, and V30, achieving high sensitivity, specificity, and accuracy in both the training and validation sets. Currently, there is no radiomics prediction of radiotherapy adverse reactions based on MRI.

#### 3.3.4 Recurrence and metastasis prediction

For early-stage liver cancer, surgical resection stands as the primary treatment modality. Nonetheless, the substantial recurrence rate within five years post-tumor excision underscores the persistent challenge of recurrence as a leading cause of postoperative mortality. Anticipating postoperative recurrence in HCC patients before surgery holds promise for enhancing the identification of high-risk individuals, thereby refining surgical strategies and clinical decision-making processes. Gao et al. (60) encompassed 472 HCC patients and developed a deep-learning predictive model based on multiphasic MRI radiomics features to predict early recurrence after HCC surgery. The integrated model yielded an impressive area under the curve (AUC) scores of 0.911 and 0.840, with accuracies of 0.779 and 0.777, sensitivities of 0.927 and 0.769, and specificities of 0.720 and 0.779 in the training and validation cohorts, respectively. These findings substantiate the effectiveness of MRI radiomics in non-invasively pinpointing high-risk individuals prone to early recurrence post-liver resection for HCC.

The results demonstrated that the models developed by Zhao et al. (61) for Clinical-Radiomics (CR), Radiomics combined with Clinical-Radiomics (RCR), and Deep Learning combined with RCR (DLRCR) could predict the recurrence of HCC. With AUC, accuracy, sensitivity, and specificity of 0.917, 0.886, 0.889, and 0.882 in the training cohort and 0.844, 0.818, 0.800, and 0.846 in the validation cohort, respectively, the DLRCR model outperformed all other models.

Furthermore, Li et al. (62) and Cao et al. (63) constructed predictive models for early recurrence after HCC surgery by integrating clinical predictors and MRI radiomics features, which are expected to become effective tools. Notably, Glypican-3 (GPC3) is an independent risk factor for postoperative recurrence of HCC. Chong et al. (64, 65) revealed the utility of preoperative Gd-EOB-DTPA MRI radiomics models in predicting GPC3-positive expression and associated recurrence-free survival (RFS) in ≤ 5 cm hepatocellular carcinoma, signifying their potential as preoperative biomarkers for early recurrence in HCC patients devoid of major vascular invasion.

For patients with hepatocellular carcinoma (HCC) treated with conventional transarterial chemoembolization (c-TACE), preoperative assessment of HCC recurrence and metastasis is also crucial for subsequent follow-up and treatment strategizing. Song et al. (66) devised an integrated model merging radiomics and clinical predictors via LASSO Cox regression, univariate and multivariate Cox regression, and Kaplan-Meier analysis to assess recurrence-free survival (RFS) post-c-TACE treatment among HCC patients. Peng et al. (67) formulated and validated radiomics machine learning (Rad-ML) models predicated on preoperative MRI to prognosticate extrahepatic metastasis (EHM) in HCC patients who have undergone transarterial chemoembolization (TACE). Among them, the XGBoost-based Rad-ML model exhibited superior predictive performance for EHM, poised to serve as a valuable asset in HCC metastasis prediction.

### 3.4 Challenges in the application of MRI radiomics in hepatocellular carcinoma

Although MRI radiomics has shown great potential in the application of hepatocellular carcinoma, it still faces a series of challenges in actual clinical application.

#### 3.4.1 Imaging acquisition and standardization

Different imaging equipment and parameters may lead to inconsistent data quality, requiring the establishment of unified data acquisition and preprocessing standards to ensure the comparability of data across institutions and studies. Data collection must comply with the ACR–AAPM–SIIM TECHNICAL STANDARD FOR THE ELECTRONIC PRACTICE OF MEDICAL IMAGING. It is essential to have physicians and radiographers who have undergone specialized training. The DICOM Standard is to be used for image transactions with the image management system. The DICOM (Digital Imaging and Communications in Medicine) standard is a protocol that describes how medical images and their metadata are stored and transmitted between devices. The DICOM standard ensures compatibility between different devices and systems, enabling seamless sharing of medical images within and between hospitals. It provides significant technical support for the healthcare sector, enhancing the efficiency and quality of diagnosis and treatment (68).

Additionally, some scholars have proposed that when applying machine learning and deep learning, since the quality of data is heavily relied upon, it is best to adhere to the METRIC framework. The METRIC framework primarily emphasizes Measurement Process, Timeliness, Representativeness, Informativeness, and Consistency. The intention of the METRIC framework is to assess the appropriateness of a dataset with respect to a specific use case (69).

#### 3.4.2 Image segmentation and feature stability

Radiomics features are not only influenced by image acquisition but also by the delineation of the region of interest (ROI). Therefore, it is essential to have experienced physicians to outline the region of interest, ensuring accuracy and reproducibility. Many studies have shown that the use of automatic or semi-automatic techniques can reduce human error. However, there is a variety of ROI delineation and feature extraction software, and currently, to address the consistency issues between different software, Zwanenburg et al. presented the Image Biomarker Standardization Initiative (IBSI), comprising a uniform set of 169 standardized features. This initiative streamlines the validation and calibration processes for diverse radiomics software tools, tackling the disparities and inconsistencies in radiological feature extraction methodologies utilized across various studies and clinical environments. Building upon this foundation, it is anticipated that more comprehensive standards will be established in the future (70, 71).

#### 3.4.3 Model generalizability and explainability

Tumor heterogeneity in HCC may affect the predictive accuracy of the model, so it is important to consider this while constructing the model. At present, most radiomics analysis studies are single-center retrospective studies, and there are significant differences between scanning equipment, parameters, and analysis software, as a result, the constructed predictive models need internal and external validation to ensure their generalizability in different populations and clinical settings.

Radiomics models based on DL are often perceived as “black boxes” by clinicians, accurately predicting specific clinical outcomes but lacking interpretable explanations. To tackle this challenge, ongoing developments in radiomics techniques aim to merge the strengths of DL with the interpretability provided by hand-crafted methods (72).

#### 3.4.4 Technological updates

With the rapid development of imaging technology and analysis methods, existing radiomics models may quickly become outdated, requiring continuous research and development. Currently, deep learning is a popular trend, but undoubtedly, new trends will emerge in the future. Despite these challenges, the use of MRI radiomics in liver cancer research is still advancing, and we anticipate overcoming these barriers as technology and clinical experience develop.

## 4 Conclusion

This review summarizes the general workflow of radiomics and the current popular deep learning methods, providing an overview of the diagnostic and differential capabilities, as well as the predictive abilities in pathological grading, treatment response, and prognosis of models based on MRI radiomics and artificial intelligence in hepatocellular carcinoma (HCC). Additionally, we discuss some of the challenges and limitations of radiomics in clinical applications, including the standardization of steps such as image acquisition and feature extraction, the generalizability of radiomics models, and the interpretability of deep learning models.

Overall, radiomics assists in analyzing the relationship between high-dimensional quantitative imaging features and clinical data, serving as a powerful tool for making personalized treatment decisions for patients. To secure the prospective clinical utilization of these models, additional investigative efforts are essential for affirming their efficacy and for bolstering their interpretive clarity. It is imperative to conduct prospective, extensive, and multicenter trials prior to their clinical deployment. Utilizing sophisticated computational methodologies, the processes preceding and succeeding the model development must incorporate clinical and multifaceted data to enhance the models’ interpretability.

## Data Availability

The original contributions presented in the study are included in the article/supplementary material. Further inquiries can be directed to the corresponding author.
